# What is the “washout” of hepatocellular carcinoma as observed on the equilibrium phase CT?: consideration based on the concept of extracellular volume fraction

**DOI:** 10.1007/s11604-022-01295-w

**Published:** 2022-06-10

**Authors:** Keiko Sakamoto, Shinji Tanaka, Keisuke Sato, Emi Ito, Marie Nishiyama, Hiroshi Urakawa, Hisatomi Arima, Kengo Yoshimitsu

**Affiliations:** 1 Department of Radiology, Faculty of Medicine, Fukuoka University, 7-45-1 Nanakuma, Jonanku, Fukuoka, Japan; 2 Department of Preventive Medicine and Public Health, Faculty of Medicine, Fukuoka University, 7-45-1 Nanakuma, Jonanku, Fukuoka, Japan

**Keywords:** Hepatocellular carcinoma, Washout, Equilibrium phase CT, Extracellular volume fraction

## Abstract

**Purpose:**

To verify the hypothesis that extracellular volume fraction (ECV) and precontrast CT density are the main determinants of washout of hepatocellular carcinoma (HCC) at the equilibrium phase CT.

**Materials and methods:**

Between 2018 and 2020, patients with surgically resected HCC were recruited who had undergone preoperative 4-phase CT. Those larger than 6 cm were excluded to minimize the possibility of intratumoral hemorrhage or degeneration. Two radiologists reviewed the whole images in consensus and divided cases into washout positive and negative groups. Washout positive group at the equilibrium phase was defined as “HCC showing relatively low density as compared to the surrounding background liver (BGL), irrespective of the presence of early enhancement or fibrous capsule”. Several clinico-pathological and radiological features, including ECV and precontrast CT density, were correlated to the presence of washout, using uni- and multi-variable analyses.

**Results:**

27 HCC in 24 patients met the inclusion criteria. 22 (82%) and five HCC belonged to washout positive and negative groups, respectively. Univariable analysis revealed ECV of HCC and BGL, ECV difference between HCC and BGL, and presence of fibrous capsule on the equilibrium phase CT were the significant factors. Multivariable analysis showed ECV of HCC and BGL, and precontrast CT density of BGL, were the independently significant factors related to washout, suggesting washout is more likely observed with lower HCC ECV, higher BGL ECV, and higher BGL precontrast CT density.

**Conclusion:**

Major determinants of washout of HCC may be ECV of HCC and BGL, and precontrast CT density of BGL.

## Introduction

Major CT features of hepatocellular carcinoma (HCC) include arterial phase hyperenhancement (APHE) and washout (WO) at the portal venous and/or delayed phase, which have been well established and well described, and defined in Liver Reporting and imaging Diagnosis System (LI-RADS) 2018 [1]. As for the former, extensive investigations have been elaborated so far, revealing that APHE is a consequence of increased tumor neovascularity during multistep hepatocarcinogenesis [2], however, little studies have been done for the latter. One study has reported that WO at the portal venous phase may be related to the thickness of tumor trabecula, sinusoidal space, and histological grade of HCC [3], but the mechanism of WO at the delayed or equilibrium phase has barely been investigated.

Recently, extracellular volume fraction (ECV) of the liver has drawn attention as a biomarker of liver fibrosis, which can be easily calculated from non-contrast and equilibrium phase CT data [4–8]. ECV is a sum of intravascular and extravascular extracellular spaces, and is simply expressed as (100 − hematocrit) * Δ liver/Δ blood pool (%), where Δ represents the difference in the CT values between at the precontrast and equilibrium phase, because the concentration of iodine is considered the same for both intra- and extra-vascular spaces at the equilibrium phase [4–8]. We have recently reported the usefulness of ECV map, which is generated by subtracting precontrast images from equilibrium phase images utilizing non-linear non-rigid anatomical correction algorithm specifically adjusted to upper abdominal organs [8, 9], and promising results have been obtained for the estimation of degree of liver fibrosis [8]. Thanks to the highly accurate subtraction algorithm, the anatomical misregistration is minimized in this ECV map, and therefore precise ECV can be obtained for any small area of any part of the upper abdomen.

**Fig. 1:**
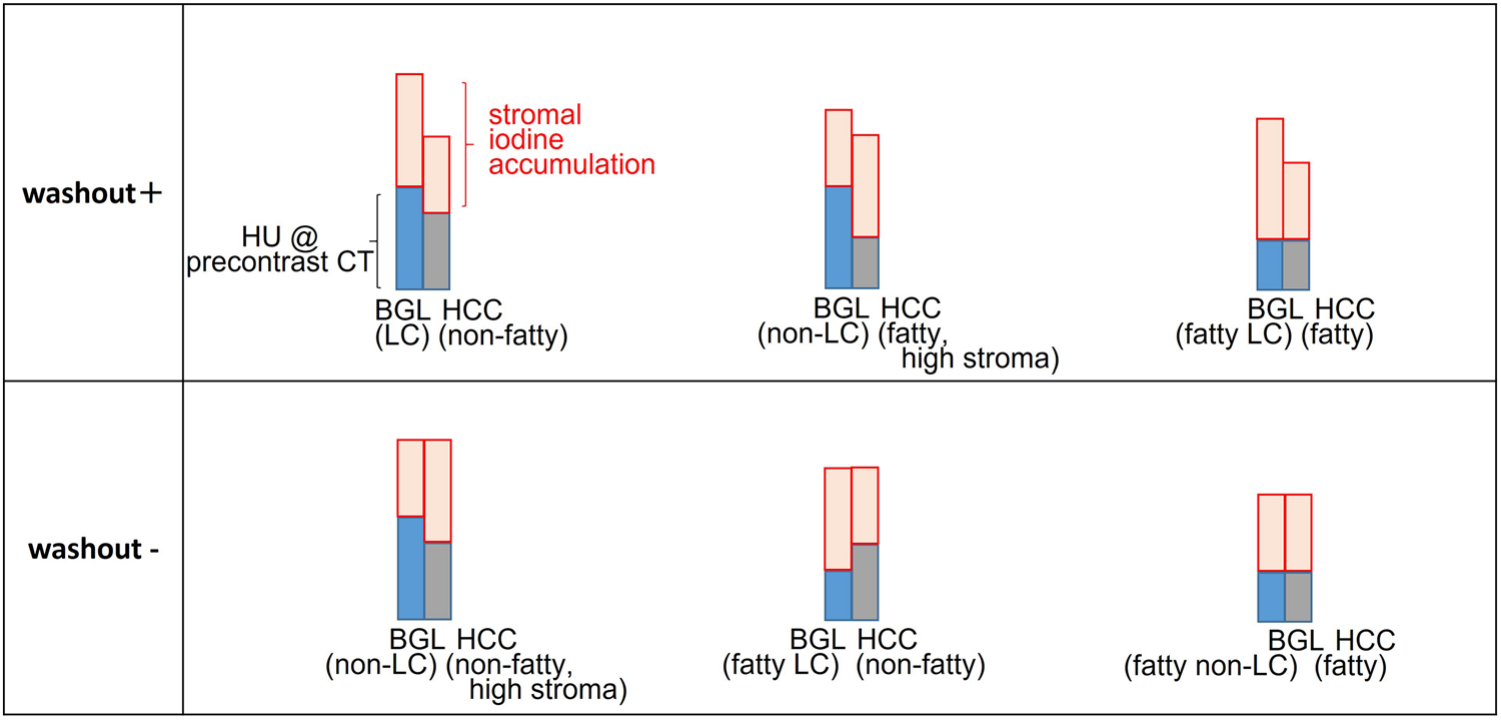
Schematic presentation of various patterns of washout status in correlation with precontrast density of and iodine accumulation in hepatocellular carcinoma (HCC) and background liver (BGL). *HU* Hounsfield unit, *LC* liver cirrhosis. The height of the bars indicates the density of HCC and BGL at the equilibrium phase. Precontrast density would be low when fatty change is present either for BGL or HCC. When BGL is cirrhotic (LC), stromal iodine accumulation would be high in BGL. When fibrotic component or sinusoidal dilatation is present, stromal iodine accumulation would be high in HCC. We hypothesized washout status would be determined by relative relationship of the precontrast density and degree of iodine accumulation between HCC and BGL at the equilibrium phase

Theoretically, WO represents the relative lowness of the sum of precontrast CT density and iodine accumulation at the equilibrium phase of HCC as compared to that of background liver (BGL) (Fig. 1). Because ECV is a standardized index for iodine accumulation in tissues, we hypothesized that precontrast densities and ECV of HCC and BGL (pre-density_HCC_, pre-density_BGL_, ECV_HCC_, and ECV_BGL_), or their differences (Δpre-density and ΔECV) may be major determinants for the WO status of HCC, and conducted this retrospective study using ECV map to clarify the mechanism of WO of HCC at the equilibrium phase CT.

## Subjects and methods

### Patients

Between January 2018 and December 2020, there were 32 consecutive surgically resected HCC patients, all of whom had undergone 4-phase preoperative CT. Among these, HCC with the largest diameter less than 6 cm were selected to minimize the chance of intratumoral degeneration or necrosis, for adequate ECV measurement. Obtaining informed consent for this study was waived by our institutional review board due to its retrospective nature.

### CT protocol

Two types of CT equipment were used. One was an area-detector CT (Aquilion ONE ViSION Edition, Canon Medical Systems), and scanning parameters were as follows: 0.5 mm × 80 row, 120 kVp, three-dimensional auto-exposure control (Volume EC: SD12@5 mm), 0.5 s/rotation, 0.813 beam pitch, 512 × 512 matrix, 300–350-mm field-of-view, and 1- or 2-mm reconstruction. Noise reduction was achieved by a hybrid iterative reconstruction (Adaptive Iterative Dose Reduction or AIDR 3D Weak). Second CT was Aquilion 64 (Canon Medical Systems), and parameters were as follows; 0.5 mm × 64 row, 120 kVp, three-dimensional auto-exposure control (Volume EC: SD12@5 mm), 0.5 s/rotation, 0.828 beam pitch, 512 × 512 matrix, 300–350-mm field-of-view, and 2-mm reconstruction (filtered back projection).

After obtaining precontrast images, 600 mgI/kg iodine contrast medium (Iopamiron 370, Bayer Health Care) was injected for 30 s at a variable injection rate, and arterial dominant phase images were obtained using bolus tracking method, followed by portal dominant phase at 60 s, and equilibrium phase images at 240 s after the commencement of contrast medium injection.

### Assessment

One of the authors (KS) generated ECV map on the dedicated workstation according to the previously reported method [8, 9], and placed as large a circular or free-hand region-of-interest (ROI) as possible for HCC and adjacent BGL, referring to original four-phase CT images. For HCC, areas of apparent fibrous capsule and necrosis was avoided; for BGL, apparent vessels were avoid for ROI placement. The second author (ST) independently measured ECV on the ECV map, and thus the inter-rater agreements were assessed. These two authors independently measured CT values (density) of HCC and BGL on the precontrast CT as well.

These two authors also reviewed all CT images including the precontrast and equilibrium phases for all HCCs on picture archiving and communication system (RapideyeCore, Canon Medical Systems, Tokyo) with a window level/width of 55/350, and independently assessed the presence or absence of fat, fibrous capsule (FC), HCC density as compared to BGL (low/slightly low/iso), and WO. Fat was defined as areas lower than 0 Hounsfield unit (HU) on precontrast CT; FC was defined peripheral high density rim on the equilibrium phase images. For the assessment of WO, readers were asked to interpret equilibrium phase images alone, and WO positivity was defined as relatively low density of HCC as compared to the surrounding liver tissue, irrespective of the presence of early enhancement or FC. The first author (KS) reviewed the electric charts of the patients, and relevant clinical data, including etiology of the chronic liver disease and Child–Pugh scores, were recorded. The same author reviewed pathological reports and several pathological findings related to HCC were recorded as well.

The presence or absence of WO was correlated to clinico-pathological factors and CT findings including precontrast densities and ECV, to elucidate significant factors related to the presence of WO. Sub-analysis was further performed to analyze the obtained results for clarification.

### Statistical analysis

As for quantitative variables (ECV and density measurement), inter- and intra-rater agreement between the two radiologists was assessed by intraclass correlation coefficients, and the averaged value between the two radiologists were adopted for statistical assessment. As for qualitative variables (CT features), kappa values were calculated for agreement assessment between the two radiologists, and disagreement was resolved by consensus. Univariable analysis was performed with *t* test or Fisher’s exact probability test for parametric variables, and with chi-square test, Mann–Whitney test, or Wilcoxon’s rank test, for non-parametric variables. Before performing multivariable analysis, multicollinearity was considered to be present when correlation coefficient was greater than 0.4 among the variables, and either one of them was discarded. Then, multivariable analysis was performed for factors related to our hypothesis (ECV_HCC_, ECV_BGL_, ΔECV, pre-density_HCC_, pre-density_BGL_, and Δpre-density) and significant ones at univariate analysis using nominal logistic regression method. *p* values less than 0.05 was considered as statistically significant. All statistical analyses were performed using JMP^Ⓡ^ 14.3.0 (SAS corporation).

## Results

There were 27 HCC in 24 patients which met the inclusion criteria. WO was present in 22 HCC and 5 HCC showed no WO. 5 HCC without WO exhibited isodensity as compared to the surrounding liver. As for the assessment of WO status, there was no disagreement between the two radiologists (*κ* value 1.0).

**Fig. 2:**
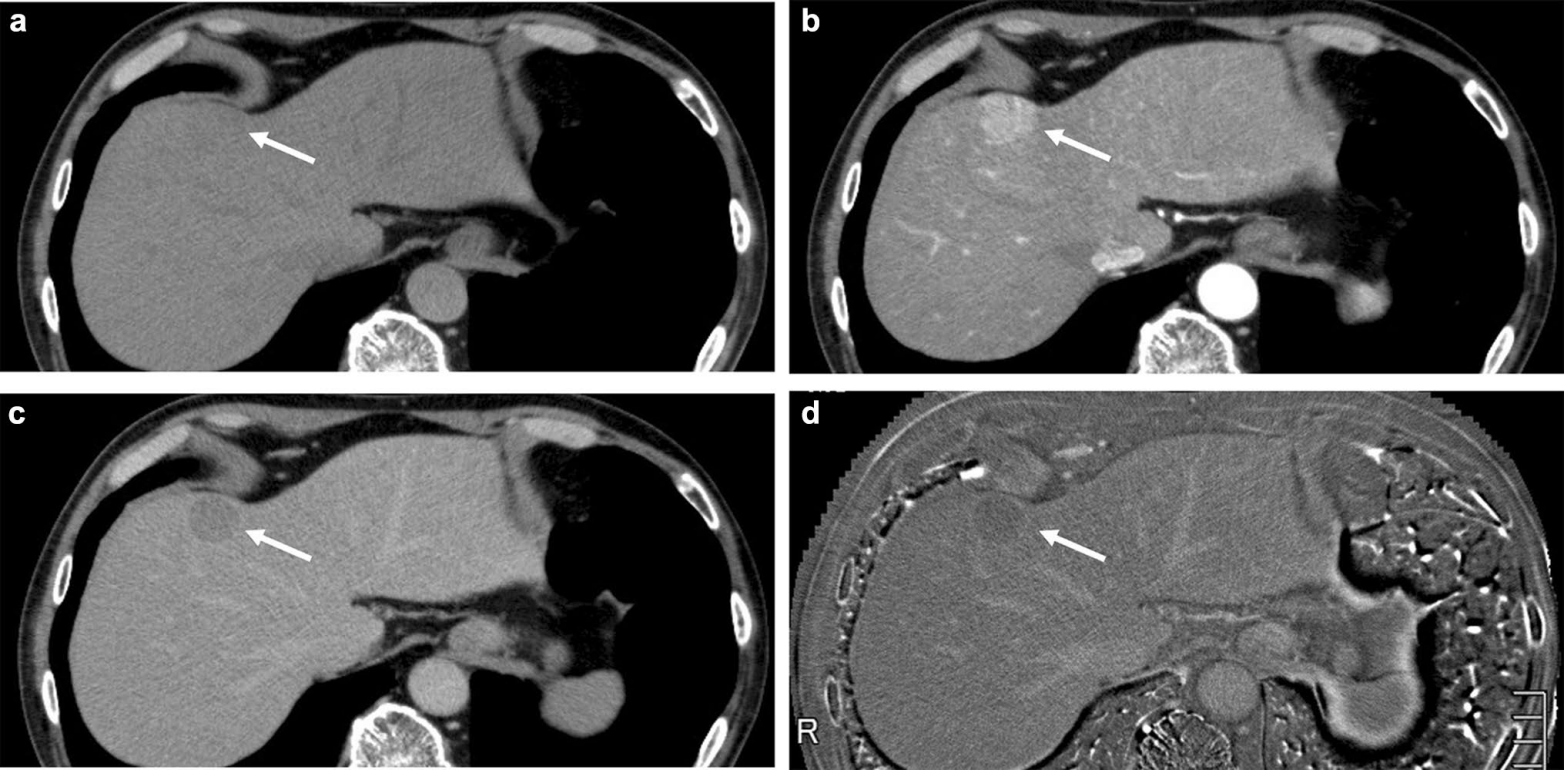
63 year-old man with chronic hepatitis C infection. Pathologically, a well differentiated hepatocellular carcinoma, mainly consisting of trabecular component, associated with fibrous capsule, was diagnosed. No septal formation or fatty change was evident. **a** Precontrast CT. The tumor exhibits almost iso- to minimally low density (arrow). Measured density were 55 and 59 Hounsfield units for the tumor and the background liver, respectively. **b** Arterial phase CT. The tumor shows homogeneous enhancement (arrow). **c** Equilibrium phase CT. The tumor exhibits apparent “washout” (arrow). **d** Extracellular volume fraction (ECV) map reveal apparently lower value of the tumor, as compared to the background liver. Measured ECV were 20.2 and 26.1% for the tumor and the background liver, respectively. In this case, although the precontrast density of the tumor or the liver were almost identical, it was considered apparently lower ECV of the tumor contributed to the positive washout

**Fig. 3:**
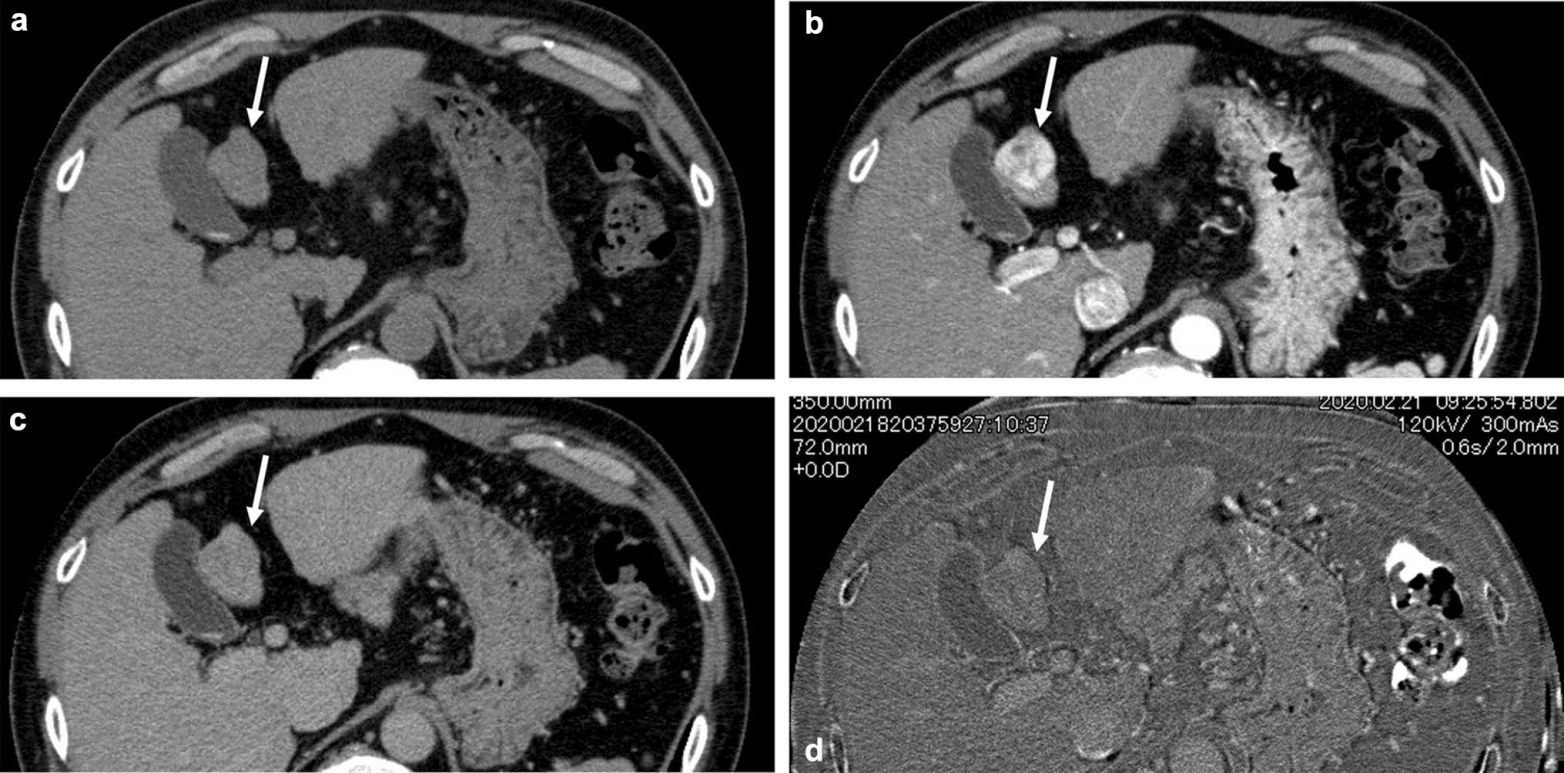
57-year-old man with chronic hepatitis B infection and habitual alcohol over-intake. Pathologically, a well differentiated hepatocellular carcinoma, mainly consisting of compact component, associated with fibrous capsule, was diagnosed. Within the tumor, apparent septal formation was seen, but no fatty change was evident. **a** Precontrast CT. The tumor exhibits almost iso- to minimally lower density than the background liver (arrow). Measured densities were 50 and 54 Hounsfield units for the tumor and the background liver (segment 4), respectively. **b** Arterial phase CT. The tumor shows heterogeneous enhancement (arrow). **c** Equilibrium phase CT. The washout of the tumor is not apparent, showing heterogeneous appearance (arrow). **d** Neither on extracellular volume fraction (ECV) map, the tumor is hard to be recognized. Measured ECV were 33.5 and 26.8% for the tumor and the background liver, respectively. In this case, although the precontrast density of the tumor or the liver were almost identical, it was considered higher ECV of the tumor obscured the apparent washout

As for variables, four clinical, eight pathological, and nine radiological (CT) factors were assessed (Table 1). The kappa values between the two radiologists for qualitative variable assessments ranged from 0.83 to 0.92, suggesting excellent agreement. Intra-class correlation coefficients for quantitative variable assessments ranged from 0.81 to 0.95, also suggesting excellent agreement. Among these variables, correlation coefficients between ECV_HCC_ and ΔECV (ECV_BGL_–ECV_HCC_), and between ECV_BGL_ and ΔECV, were − 0.82 and 0.53, respectively (Table 2), and therefore ECV_HCC_ and ECV_BGL_ were adopted as variables representing these three factors, considering the positive multicollinearity. Similarly, correlation coefficients between precontrast density of HCC (pre-density_HCC_) and Δpre-density (pre-density_BGL_ − pre-density_HCC_), and between pre-density_BGL_ and pre-density_HCC_, were − 0.93 and 0.46, respectively (Table 2), and therefore pre-density_BGL_ and Δpre-density were adopted as variables representing these three factors. Univariable analysis revealed presence of FC at the equilibrium phase CT, ECVs of HCC and BGL as significant factors, and pathological presence of intratumoral fat was marginal (*p* = 0.05) (Table 1). These three significant and one marginal factors, and pre-density_BGL_ and Δpre-density were input to nominal logistic regression analysis, revealing ECV_HCC_, ECV_BGL_, and pre-density_BGL_ were independently significant factors (Table 3). Namely, the smaller ECV_HCC_ is, and the larger ECV_BGL_ and pre-density_BGL_ are, HCC are more likely to show WO on the equilibrium phase CT. Representative cases are shown in Figs. 2 and 3.

**Table 1:** Clinico-pathological and radiological factors vs washout status

	Washout + (*n* = 22)	Washout − (*n* = 5)	*p* value	ICC/*κ* value
Clinical factors				
Age	73.9 ± 9.0 (*n* = 20)	71.3 ± 9.8 (*n* = 4)	NS (0.73)	
Sex (m/f)	17/3 (*n* = 20)	2/2 (*n* = 4)	NS (0.17)	
Chronic liver disease (B/C/NBNC)	3/13/6	2/1/1	NS (0.06)	
Child–Pugh score (5/6)	22/0	5/0	NS (1.00)	
Pathological factors				
Tumor size (mm)	29.4 ± 13.2	23.8 ± 9.6	NS (0.64)	
Macroscopic type (NiM/SN/MN)	2/17/3	1/3/1	NS (0.72)	
Histological grades (e/w/m/p)	2/8/9/3	1/3/1/0	NS (0.70)	
Growth pattern (tr/pg/c/s)	14/1/7/0	4/0/1/0	NS (0.71)	
Scirrhous subtype^a^ (p/n)	20/2	5/0	NS (0.42)	
Septal formation (p/n)	12/10	3/2	NS (0.83)	
Intratumoral steatosis (p/n)	7/15	4/1	NS (0.05)	
Bile production (p/n)	3/19	1/4	NS (0.72)	
Fibrous capsule (p/n)	6/16	1/4	NS (0.73)	
BGL presence of cirrhosis (p/n)	5/17	0/5	NS (0.24)	
Radiological factors				
Precontrast density of HCC (low/slightly low/iso)	9/6/6	2/2/1	NS (0.90)	0.83
FC on equilibrium phase CT (p/n)	15/7	1/4	0.04	0.85
Intratumoral steatosis (p/n)	4/18	1/4	NS (0.93)	0.92
ECV of HCC	27.4 ± 6.5	37.6 ± 5.3	< 0.01	0.83
ECV of BGL	29.6 ± 5.1	24.3 ± 2.5	0.01	0.81
ΔECV(BGL-HCC)	27.2 ± 6.4	37.3 ± 5.9	0.01	
Precontrast HU of HCC	43.0 ± 17.9	39.6 ± 9.0	NS (0.12)	0.95
Precontrast HU of BGL	58.4 ± 5.7	56.6 ± 9.2	NS (0.46)	0.85
Precontrast ΔHU (BGL − HCC)	15.3 ± 16.0	17.0 ± 10.3	NS (0.37)	

*ICC* intraclass correlation coefficient, *m/f* male/female, *B/C* hepatitis B/C infection, *NBNC* non-B non-C, *NiM* small nodule with indistinct margin, *SN* simple nodular, *MN* confluent multinodular, *e* early HCC, *w/m/p* well, moderately, and poorly differentiated, *tr* trabecular, *pg* pseudoglandular, *c* compact, *s* scirrhous, *p/n* positive/negative, *BGL* background liver, *HCC* hepatocellular carcinoma, *FC* fibrous capsule, *ECV* extracellular volume fraction, *HU* Hounsfield unit

^a^Because there was no scirrhous dominant case, HCC with scirrhous component, if any, were included

**Table 2:** Multicollinearity assessment

Factor	Vs factor	*r*^2^	*r*	*p*-value
ECV of HCC	ECV of BGL	0.002	0.045	0.82
	ΔECV	0.67	− 0.82	< 0.0001
ECV of BGL	ΔECV	0.29	0.53	0.0041
Pre-density of HCC	Pre-density of BGL	0.21	0.46	0.016
	ΔPre-density	0.86	− 0.93	< 0.0001
Pre-density of BGL	ΔPre-density	0.0088	− 0.094	0.64

*r* correlation coefficient, *ECV* extracellular volume fraction, *pre-density* Hounsfield unit on precontrast CT, *HCC* hepatocellular carcinoma, *BGL* background liver

**Table 3:** Nominal logistic regression analysis

Factor	*p*-value	Unit odds ratio (95% CI)^a^	Range odds ratio (95% CI)^a^
ECV_HCC_	0.00016	1.30 (1.05–1.62)	2174.0 (3.72–1,269,606)
ECV_BGL_	0.00027	0.76 (0.55–1.06)	0.0043 (5.87 e^−6^–3.12)
Pre-density_BGL_	0.0024	0.94 (0.80–1.11)	0.22 (0.0039–12.66)
FC on CT	1.00		
ΔPre-density	1.00		
Intratumoral steatosis	1.00		

*CI* confidence interval, *ECV* extracellular volume fraction, *HCC* hepatocellular carcinoma, *BGL* background liver, *pre-density* Hounsfield unit on precontrast CT, *FC on CT* fibrous capsule on equilibrium phase CT, *Δdensity* the difference in density between HCC and BGL, *intratumoral steatosis* pathological fatty change of HCC

^a^Unit and range odds ratios were calculated by univariable logistic regression analysis, because multivariable model did not converge, probably due to small number of washout negative cases

**Fig. 4:**
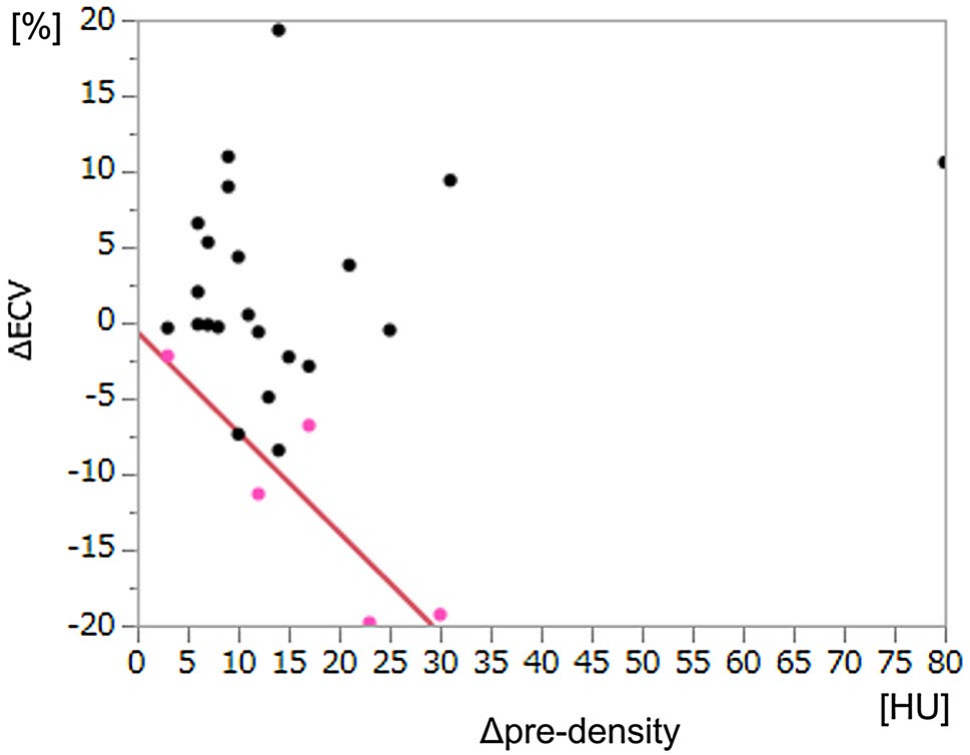
A graph with *x* and *y* axes representing Δpre-density (precontrast density of background liver − that of tumor) and ΔECV (ECV of background liver − that of tumor), respectively, for sub-analysis. Pink dots indicate hepatocellular carcinoma (HCC) without washout, for which a regression line is shown in red. Black dots indicate HCC with washout, all of which are located above the regression line

As a sub-analysis, we plotted all cases on the graph with *x* and *y* axes representing Δdensity and ΔECV, respectively (Fig. 4). First, a regression line was drawn for the five HCCs which showed no WO, in other words, exhibiting iso-density as the BGL at the equilibrium phase CT. This line therefore indicates where the effect of CT density elevation difference due to iodine accumulation (ΔECV) is just cancelled or balanced by the difference in precontrast densities between HCC and BGL (Δpre-density). The area above this line (right upper hand) represents where equilibrium phase density of HCC is lower than that of BGL, namely WO is positive, either by lower ΔECV or by higher Δpre-density. On the other hand, the area below this line (left lower hand) represents where equilibrium phase density of HCC is higher than that of BGL, namely, WO is negative, showing delayed or prolonged enhancement, although there was no such case in our present cohort. As shown in Fig. 4, all HCC with positive WO were virtually above that line.

## Discussion

As we hypothesized, multivariable analysis showed ECV_HCC_, ECV_BGL_, and precontrast density of BGL, were independently significant factors related to WO status of HCC. In other words, the smaller ECV_HCC_ is, and the larger ECV_BGL_ and pre-density_BGL_ are, the more likely WO is observed on equilibrium phase CT. The reason why pre-density_BGL_ was not significant on univariable analysis may be small number of subjects in the current study. Similarly, the reason why pre-density_HCC_ was not significant on both uni- and multi-variable analyses may also be attributable to small number of subjects. The negative influence of steatosis in BGL on the WO status, namely, that WO of HCC may be obscured by the presence of BGL steatosis, has recently been reported by several investigators [10, 11] and is concordant to our results.

One may argue that because both washout status (objective variable) and ECV (explanatory variable) are determined by the precontrast and equilibrium phase densities, our results may be self-evident. It is true, that both variables are related to the same CT densities, but actually are determined by completely different ways; the former (washout status) is determined by whether the sum of precontrast density and degree of iodine accumulation is less than that of BGL (Fig. 1), whereas the latter (ECV) is determined by the formula as shown in the Introduction section, being an index of iodine accumulation standardized by several physiologic factors including renal function, body mass index, degree of anemia, and also by dosage of contrast agent. In other words, ECV and washout are related to each other in some extent, but are two different variables, because there are several unknown factors present to predict one from the other. That is the reason why we needed to carry out this validation study.

In the daily clinical practice, we often experience difficulty in recognizing WO in small HCCs, but in the current study, tumor size was not a significant factor to discriminate WO+ vs WO− group, which at least in part would be attributable to small number of WO− cases in our study population.

As for the delay time of “equilibrium” phase, we have adopted 4 min since 2008, which is slightly longer than more common 3 min, because we felt it would provide better delineation of prolonged or delayed enhancement of hemangioma or cholangiocellular carcinoma than 3 min, which are major important differential diagnoses of HCC. We believe this 4-min delay time is more adequate, not to say optimal, than 3 min, for the calculation of ECV [8]. This is the reason why we used the term “equilibrium phase” instead of “delayed phase” throughout this manuscript.

In this study, none of pathological features of HCC were related to WO status at multivariable analysis, although intratumoral steatosis and FC as observed on CT (radiological FC) were suggested to be marginally and significantly related to WO positivity at univariable analysis, respectively. Because fatty change or intratumoral steatosis of HCC would lower the density of HCC, it may reasonably lead to WO positivity. The reason why intratumoral steatosis was not a significant factor at multivariable analysis may at least partly be attributable to the small number of subjects in our study, but recent investigations with larger cohort also reported similar results [10]. Fibrous capsule (FC) was pathologically observed in 7 out of 27 tumors (26%), which was not related to WO status, on the other hand, radiological FC was recognized in 16 out of 27 tumors (59%), which was significantly related WO positivity at univariate analysis. This discrepancy may be explained as follows: radiological FC as observed on the equilibrium phase CT is considered to represent complex of true or pathological FC and surrounding compressed liver parenchyma with sinusoidal dilatation, which would retain iodine contrast at the equilibrium phase CT [12]. This compressed liver parenchyma with dilated sinusoids alone, therefore, even without true or pathological FC, may result in hyperdense rim, namely FC appearance, explaining the higher incidence of radiological FC (59%) than that of pathological FC (26%). This FC appearance has been reported to have illusional effects as if WO was present even though the densities inside and outside of FC is identical [13]. This is in concordance with recent report [10]. Scirrhous type HCC is a rare subtype of HCC, accounting for 5% of all HCC, characterized by its abundant stromal fibrosis [14, 15]. This stromal fibrosis has been reported to cause delayed or prolonged enhancement on CT [15], which should be negatively related to WO status. In our series, however, there was no typical scirrhous HCC, and only two cases had scanty scirrhous components, both of which showed clear WO. Another subtype of HCC which would theoretically show delayed or prolonged enhancement, obscuring WO, is HCC with substantial peliotic change [16, 17]. There was no such case in our patient group either.

Recently, Mehara et al. [18] have published a meticulous pathological report on the intratumoral fibrosis of HCC. They revealed that there are significant amount of collagen and elastin within HCC, which are closely related to various histopathological and immunohistochemical features of HCC, some of which have been shown to be associated with biological aggressiveness of HCC and consequently, patient prognosis [18]. As mentioned earlier, ECV is a sum of intravascular and extravascular extracellular spaces, and the latter is where intratumoral fibrosis of HCC is present; because the former (intravascular or sinusoidal space) has been reported to be almost constant regardless of the histological grades of HCC [8], ECV could potentially be a good biomarker to represent intratumoral fibrosis, which might predict biological aggressiveness of HCC, and hopefully patient prognosis as well.

There are several limitations present in our study. First, the total number of subjects is small, particularly that of WO negative tumors, which could have influenced the current results as mentioned above. Second, pathological specimens were not reviewed specifically for this study, and simply pathological reports, made by one expert pathologist, were referred to for pathological feature assessment. Third, we used two different CT scanners, which could have affected the results. Fourth, generation of ECV map is not available at all institutions. Finally, because of its retrospective nature, some biases could not be completely excluded.

In conclusion, WO of HCC on the equilibrium phase CT is likely to be determined by ECV of HCC and BGL, and also by precontrast density of BGL. Radiologists should be aware of this issue when interpreting CT images.

## References

[1] https://www.acr.org/Clinical-Resources/Reporting-and-Data-Systems/LI-RADS/CT-MRI-LI-RADS-v2018. Lastly Accessed 8 Jan 2022.

[2] Matsui O, Kobayashi S, Sanada J (2011). Hepatocellular nodules in liver cirrhosis: hemodynamic evaluation (angiography-assisted CT) with special reference to multi-step hepatocarcinogenesis. Abdom Imaging.

[3] Okamoto D, Yoshimitsu K, Nishie A (2012). Enhancement pattern analysis of hypervascular hepatocellular carcinoma on dynamic MR imaging with histopathological correlation: validity of portal phase imaging for predicting tumor grade. Eur J Radiol.

[4] Varenika VJ, Fu YJ, Maher JJ (2013). Hepatic fibrosis: evaluation with semiquantitative contrast-enhanced CT. Radiology.

[5] Zissen MH, Wang ZJ, Yee J, Aslam R, Monto AM, Yeh BM (2013). Contrast-enhanced CT quantification of the hepatic fractional extracellular space: correlation with diffuse liver disease severity. AJR.

[6] Bandula S, Punwani S, Rosenberg WM (2015). Equilibrium contrast-enhanced CT imaging to evaluate hepatic fibrosis: initial validation by comparison with histopathologic analysis. Radiology.

[7] Yoon JH, Lee JM, Klotz E (2015). Estimation of hepatic extracellular volume fraction using multiphasic liver computed tomography for hepatic fibrosis grading. Investig Radiol.

[8] Shinagawa Y, Sakamoto K, Sato K (2018). Usefulness of new subtraction algorithm in estimating degree of liver fibrosis by calculating extracellular volume fraction obtained from routine liver CT protocol equilibrium phase data: preliminary experience. EJR.

[9] Tani T, Sato K, Sakamoto K (2021). Importance of extracellular volume fraction of the spleen as a predictive biomarker for high-risk esophago-gastric varices in patients with chronic liver diseases: a preliminary report. Eur J Radiol.

[10] Hwang JA, Min JH, Kang TW (2021). Assessment of factors affecting washout appearance of hepatocellular carcinoma on CT. Eur Radiol.

[11] Min JH, Kang TW, Kim YY (2021). Vanishing washout of hepatocellular carcinoma according to the presence of hepatic steatosis: diagnostic performance of CT and MRI. Eur Radiol.

[12] Ishigami K, Yoshimitsu K, Nishihara Y (2009). Hepatocellular carcinoma with a pseudocapsule on gadolinium-enhanced MR images: correlation with histopathologic findings. Radiology.

[13] Sofue K, Sirlin CB, Allen BC (2016). How reader perception of capsule affects interpretation of washout in hypervascular liver nodules in patients at risk for hepatocellular carcinoma. J Magn Reson Imaging.

[14] Kim SH, Lim HK, Lee WJ (2009). Scirrhous hepatocellular carcinoma: comparison with usual hepatocellular carcinoma based on CT-pathologic features and long-term results after curative resection. EJR.

[15] Hatano M, Ojima H, Masugi Y (2019). Steatotic and nonsteatotic scirrhous hepatocellular carcinomas reveal distinct clinicopathological features. Hum Pathol.

[16] Honda H, Kaneko K, Maeda T (1997). Small hepatocellular carcinoma on magnetic resonance imaging. Relation of signal intensity to angiographic and clinicopathological findings. Investig Radiol.

[17] Nomura Y, Nakashima O, Kumabe T (2014). Clinicopathologic analysis of simple nodular type of well-differentiated hepatocellular carcinoma with extensive peliotic change. JGH.

[18] Mehara J, Masugi Y, Abe T (2020). Quantification of intratumoral collagen and elastin fibers within hepatocellular carcinoma tissues finds correlations with clinico-patho-radiological features. Hepatol Res.

